# Single-Criterion Optimisation with Consideration of Uncertainties of the Composite Multi-Layer Slabs

**DOI:** 10.3390/ma19112384

**Published:** 2026-06-03

**Authors:** Przemysław Smela, Bartosz Miller

**Affiliations:** Department of Structural Mechanics, Faculty of Civil and Environmental Engineering and Architecture, Rzeszów University of Technology, al. Powstańców Warszawy 12, 35-029 Rzeszów, Poland

**Keywords:** sensitivity analysis, surrogate modelling, optimisation, deep neural network, composite, fundamental frequency, uncertainties

## Abstract

This paper presents a novel, efficient computational framework for the optimisation of the fundamental frequency of multi-layered composite slabs with consideration of uncertainties. The approach is based on Finite Element Method (FEM) data generation, Deep Neural Network (DNN) surrogate modelling, deterministic optimisation using the genetic algorithm (GA), Morris Sensitivity Analysis (SA), and quantile-based optimisation, including uncertainties and using the GA. Different boundary condition configurations are considered. The surrogate model is trained on FEM-generated samples and subsequently used to replace expensive modal analyses during optimisation, significantly reducing the optimisation evaluation cost for one boundary condition variant. The proposed method achieves near-identical optimal non-dimensional parameter Ω values to those reported in the literature for Bayesian Optimisation (BO), with discrepancies of less than 0.5%. To improve robustness to manufacturing tolerances, an additional uncertainty-aware optimisation is performed, in which model parameters are perturbed with normally distributed noise. By maximising the 5% quantile of the non-dimensional parameter Ω, robust optimal solutions are obtained with minimal loss in performance. Overall, the DNN-GA framework enables fast and accurate optimisation of composite laminates and provides both deterministic and robust design recommendations at a fraction of the computational cost of traditional FEM-based optimisation workflows.

## 1. Introduction

### 1.1. Deterministic Optimisation and Sensitivity Analysis

Composite materials consist of two or more constituent materials combined to achieve enhanced or specifically desired properties. They consist of the following elements: the dispersed phase and the continuous medium called the matrix [1].

Many materials known today are virtually composites [2]; hence, composites are finding applications in many areas of industry. The civil engineering sector is the primary consumer of composites. Additionally, the automobile, electrical and electronics, and mechanical industries represent significant consumers. Beyond these sectors, composites are also utilised in aerospace, marine, sports, and medical applications [1]. One of the composite products that has a wide range of applications is multi-layered slabs.

The composite structures (a.o. multi-layered slabs) are often exposed to severe vibration circumstances. In addition, the dynamic behaviour of composite structures is not yet well understood. Consequently, optimising anti-resonance performance, such as by maximising the natural frequency, has become increasingly significant in composite structural design [3]. Maximising the first natural frequency is crucial because resonance is the phenomenon that must be strongly avoided.

These matters are the subject of much research and articles. For example, in the article [3], the Layerwise Optimisation Approach (LOA) was applied to determine optimal layer sequences for a symmetrical eight-layer composite plate. The paper [4] presents the optimisation process, in which the FEM was considered in conjunction with the following three metaheuristics: GA, Repulsive Particle Swarm Optimisation with Local search and Chaotic perturbation (RPSOLC), and the Co-evolutionary Host–Parasite (CHP) algorithm. In another study [5], the Artificial Bee Colony (ABC) algorithm was applied to maximise the first natural frequency of the eight-layer composite slab. The obtained results were compared with the results shown in the article [3]. Wang et al. proposed in the article [6] the Hybrid Whale Optimisation Algorithm (HWOA) to optimise the stacking sequence of arbitrary quadrilateral composite plates. The publication [7] applies differential evolution optimisation to determine stacking sequences that maximise the natural frequency of both symmetric and asymmetric laminates. Finally, the article [8] describes Bayesian Optimisation (BO) as the other approach that enables solving the above-mentioned problem. In addition, the comparison and discussion of the results from the articles [3,5,6,8] are shown.

Apart from optimisation, sensitivity analysis (SA) [9] is also a very popular topic among scholars in the field of composite slabs. In the paper [10], the influence of Poisson’s ratio on the first frequency was analysed. The article [11] presents a sensitivity analysis of antisymmetric angle-ply laminates using partial derivatives. Liu et al. [12] present a general analytical sensitivity analysis method for composite laminated plates and shells, applicable to both classical and first-order shear deformation theories. In contrast, Khorshidi et al. [13] conduct a sensitivity analysis of vibrating rectangular laminated composite plates interacting with an inviscid fluid, employing the modified higher-order shear deformation plate theory.

The above-mentioned publications [3,4,5,6,7,8] describe the different approaches to optimisation. But there is also the other method of optimisation—the DNN + GA approach, which is used by the scholars and applied, a.o., in the articles [14,15,16,17,18] during the optimisation of shell composite cantilevers, thin-walled cylindrical shells, etc.

### 1.2. Optimisation with Consideration of Uncertainties

In practical manufacturing of composite laminates, many parameters, such as lamination angles, layer thicknesses, material properties, etc., are subject to unavoidable deviations, typically within a few units due to production tolerances. These deviations may significantly affect the dynamic performance of the structure, especially when the first natural frequency is used as a design criterion. However, the studies usually consider only deterministic optimisation of stacking sequences and do not assess the robustness of the obtained lay-ups to such perturbations.

When uncertainties are present in the design variables, optimisation based solely on deterministic performance may lead to solutions that are highly sensitive to small deviations. Therefore, robust formulations, such as quantile-based optimisation, are increasingly applied to engineering design problems. In particular, Kriging-based surrogate models combined with quantile-based objective functions have been shown to provide an efficient framework for uncertainty-aware optimisation [19]. Such approaches explicitly maximise a chosen statistical measure (e.g., Q_0.05_) rather than the nominal response, improving the robustness of the final design.

Several established approaches address the incorporation of uncertainties in composite structure optimisation. Peng et al. [20] presented an optimisation of hybrid composite structures that considers multiple-scale uncertainties. Kalantari et al. [21,22] described a hybrid multi-objective evolutionary algorithm for multi-directional carbon/glass fibre-reinforced epoxy hybrid composites, accounting for uncertainties in fibre angle and lamina thickness. Awruch et al. [23] proposed a fuzzy α-cut optimisation methodology combined with a heuristic algorithm to optimise vibration control performance of laminated composites under uncertainty. Akmar et al. [24] introduced an ant colony optimisation algorithm for multi-ply, fibre-reinforced hybrid composite structures under multiple-scale uncertainty. Shi et al. [25] developed an improved reliability analysis method for laminated composite structures that integrates uncertainties in elastic properties and strength characteristics. An et al. [26] presented a reliability-based design framework that considers uncertainties in delamination and material properties to optimise stacking sequences with high accuracy and efficiency. Furthermore, An et al. [27] proposed an optimisation approach for the stacking sequence design of laminated composite structures with uncertain but bounded parameters.

This article addresses the following gap: it combines the DNN + GA approach, mentioned in Section 1.1, and a quantile-based optimisation, where the most influential lamination angle is perturbed by normally distributed noise, and the 5% quantile of the non-dimensional frequency Ω is maximised using the DNN surrogate model.

The proposed framework demonstrates that surrogate models—DNNs—can serve as efficient substitutes for computationally expensive FEM simulations. The obtained optimisation results are consistent with those reported in the benchmark study [8], which supports the credibility of the proposed approach. Moreover, the sensitivity analysis identifies the most influential design variable affecting the analysed response. Finally, the quantile-based optimisation results show that the proposed framework can account for uncertainty in the most influential design parameter.

## 2. Materials and Methods

### 2.1. The Purposes of the Analysis

The primary purposes of this analysis, in relation to the composite multi-layered square slab, are:

- Single-criterion optimisation for the maximum fundamental frequency value;
- Sensitivity analysis;
- Optimisation with consideration of uncertainties.

### 2.2. Structure Description

**Figure 1:**
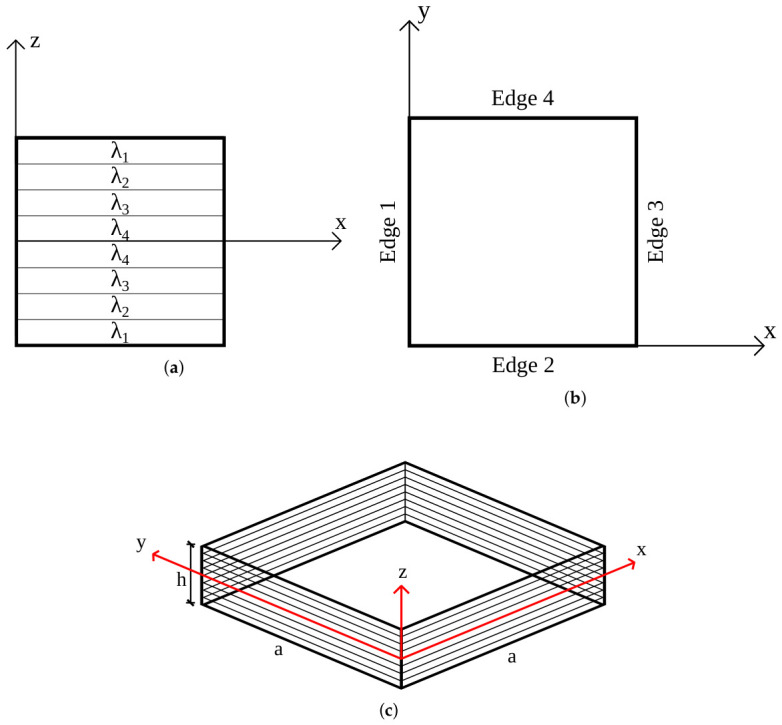
The scheme of the analysed composite slab. (**a**) Sequence of layers with indications of angles. (**b**) Numeration of edges. (**c**) 3D view of the analysed slab and the assumed coordinate system marked in red.

The analysed slab comes from Yang [8] and is shown in Figure 1.

### 2.3. Detailed Description of the Purposes of the Analysis

The first purpose of the analysis is the single-criterion optimisation problem. This problem was formulated to find the optimal set of design variables $\lambda^{*}$ (in this case, 4 lamination angles laid in the above-mentioned slab symmetrically relative to the mid-plane of the slab) that maximizes the fundamental natural frequency. The objective function is defined as:

(1) $$f_{1}=f\left(\lambda \right),$$

and the optimisation process is described in the following way:

(2) $$\lambda^{*}=\arg\min_{\lambda }\left(-f\left(\lambda \right)\right),$$

where the vector of design variables is given by:

(3) $$\lambda =[\lambda_{1},\lambda_{2},\lambda_{3},\lambda_{4}].$$

In the optimisation process, the lamination angles were restricted to a discrete set of values ranging from −$90^{\circ }$ to $90^{\circ }$ with an increment of $5^{\circ }$. This constraint can be mathematically expressed as:

(4) $$\lambda_{i}\in \left\{-90^{\circ },-85^{\circ },\ldots ,90^{\circ }\right\},\;\mathrm{for}\;i=1,2,3,4.$$

In other words, the aim of simulations is to obtain the highest possible value of the first natural frequency by optimising the lamination angles in each layer.

The lamination angle for each of the 4 layers can be chosen in 37 ways. This results in a total of 1,874,161 possible lay-ups (37^4^ possibilities), highlighting the vast design space we are dealing with. Quantifying the search space in this way underscores the problem’s complexity and justifies the need for computational approaches to efficiently navigate it.

The first natural frequency was transformed into the non-dimensional frequency parameter Ω using the following equation [8]:

(5) $$\Omega =\frac{\omega a^{2}}{\pi^{2}}\sqrt{\frac{\rho h}{D_{0}}},$$

where

(6) $$D_{0}=\frac{E_{22}h^{3}}{12(1-\nu_{12}\nu_{21})},$$

and

(7) ω=2πf.

The description of the applied parameters is collated in Table 1.

$E_{22}$, $\nu_{12}$, $\nu_{21}$, and ρ are material parameters. Their meaning and values are presented in the Table 2.

The second aim of the analysis is SA. SA allows us to point out which variable, $\lambda_{1}$, $\lambda_{2}$, $\lambda_{3}$, or $\lambda_{4}$, affects the Ω value the most.

The third and main purpose of the analysis is optimisation with consideration of uncertainties. Initially, the most influential lamination angle is selected from the SA. Then, this chosen angle is noised with uncertainties, defined as the normal distribution of a random variable [28]. Finally, the 5% quantile of Ω is maximised [29].

The objective function is defined as:

(8) $$f_{2}=f\left(\lambda +\varepsilon \right),$$

and the optimisation with the inclusion of the uncertainties process is described in the following way:

(9) $${\lambda_{j,nom}}^{★}=\arg\min_{\lambda_{j,nom}\in \Lambda }\left(-Q_{0.05}\left(\Omega \left(\lambda +\varepsilon \right)\right)\right),$$

where:

(10) $$\Lambda =\{\lambda_{j,nom}\in R:\lambda_{j,nom}\in \left[-90^{\circ },90^{\circ }\right]\},$$

(11) $$\lambda ={\left[\lambda_{j,nom},\lambda_{j,nom},\ldots ,\lambda_{j,nom}\right]}_{1\times n},\;\lambda_{j,nom}\in \Lambda ,$$

(12) $$\varepsilon ={\left[\varepsilon_{1},\varepsilon_{2},\ldots ,\varepsilon_{n}\right]}_{1\times n},\;\varepsilon_{k}∼N\left(\mu_{\varepsilon },\sigma_{\varepsilon }^{2}\right),\;k=1,\ldots ,n,$$

and

(13) $$\Omega \left(\lambda +\varepsilon \right)=[\Omega \left(\lambda_{j,nom}+\varepsilon_{1}\right),\Omega \left(\lambda_{j,nom}+\varepsilon_{2}\right),\ldots ,\Omega \left(\lambda_{j,nom}+\varepsilon_{n}\right){]}_{1\times n}.$$

Letter *j* indicates the index of the most influential angle, chosen after SA, whereas the letter *n* is the number of elements in the vectors λ and ε.

The following parameters of the normal distribution of a random variable were assumed: $\mu_{\varepsilon }$ = $0^{\circ }$ and $\sigma_{\varepsilon }$ = $2.5^{\circ }$. The mean value of the uncertainty was assumed to be $0^{\circ }$, which reflects the absence of systematic bias [28], whereas the standard deviation was assumed to be $2.5^{\circ }$, corresponding to half of the increment of $5^{\circ }$, used in the description of the deterministic optimisation. Under the assumption of a normal distribution of the $\varepsilon_{i}$, approximately 95% of the realisations of the uncertainties fall within ±2$\sigma_{\varepsilon }$, which corresponds to ±$5^{\circ }$.

### 2.4. Variants of the Boundary Conditions

During the analysis, each edge has been assigned boundary conditions. These boundary conditions are described in the form of four-letter indications. Eleven different boundary condition variants, as described in the benchmark article by Yang [8], were considered:

- CCCC;
- CCFF;
- CFCF;
- CFFF;
- SCFF;
- SCSC;
- SCSF;
- SSCC;
- SSSC;
- SSSF;
- SSSS.

Letter C (clamped) indicates that both translational and rotational degrees of freedom are fixed. In other words, the nodes located on the edge indicated by this letter can neither move nor rotate in any direction.

Letter S (simply supported) means that solely the translational degrees of freedom are fixed, and hence the rotations are allowed.

Letter F (free) means that there are no boundary conditions on this edge.

For example, the indication SCFF means that (according to Figure 1b):

- Edge number 1 is simply supported;
- Edge number 2 is clamped;
- Edge number 3 is free;
- Edge number 4 is free.

### 2.5. Methods

#### 2.5.1. Parameters of the Computational Environment

Every computation (from generating the patterns to optimisation with consideration of uncertainties) was performed in the computational server with the following parameters:

- Operating system: Rocky Linux 9.5 (Blue Onyx) 64-bit;
- Memory: 128 [GB];
- CPU: AMD^®^ Ryzen threadripper 1920X, 12-core processor × 24 (Santa Clara, CA, USA);
- Software: ANSYS^®^ Mechanical Enterprise Academic Research (Canonsburg, PA, USA), version 24.2.

#### 2.5.2. Creating the Patterns for Learning of Surrogate Models

The first part of the analysis was generating the patterns, which were used to train the surrogate model, a DNN. For this stage of analysis, in addition to the Python language, the open-source Python library PyMAPDL, version 0.70.1, which provides a programming interface to the Mechanical Ansys Parametric Design Language (MAPDL) solver, was used [30]. Combining these tools enables performing the computation without using the graphical user interface of the FEM code, ANSYS^®^.

For each variant of the boundary conditions described above, 4000 patterns were created, where the angles of lamination were chosen at random for every pattern from the discrete set of angles described in Equation (4).

For the purpose of FEM simulations, the following parameters were assumed:

- Type of finite element: SHELL 281;
- Mesh size: 0.0005 [m] (value was sourced from the article [8]);
- Number of integration points in the thickness direction: 9;
- Analysis type: modal;
- Method of computing frequencies: the block Lanczos algorithm [31].

#### 2.5.3. Creating the Surrogate Models

The second stage of the analysis was preparing the surrogate models, DNNs.

The DNNs were trained separately for each boundary condition variant. The following parameters for each neural network were assumed:

- Library: PyTorch, version 2.2.2 [32].
- Model type: Fully connected feed-forward neural network for regression.
- Number of inputs: 4 (the number of angles of lamination).
- Number of outputs: 1 (Ω value).
- Number of hidden layers: 4.
- Number of neurons in each hidden layer: 60.
- Activation function: Rectified Linear Unit (ReLU) [33];
- BatchNormalization: For each hidden layer.
- Hidden layer structure: Linear → BatchNorm1d → ReLU.
- Output layer: Linear (without activation function).
- Number of folds: 5.
- Number of epochs: 1200.
- Batch size: 512.
- Learning rate strategy: Initial learning rate: 10^−3^, warm-up phase using LinearLR (start factor = 10^−2^, 10% of epochs, minimum 5 epochs), cosine annealing schedule (CosineAnnealingLR) down to η_min_ = 10^−7^.
- Early stopping: Yes (with the following parameters: patience = 150 and Δ_min_ = 10^−4^).
- Ratio training set/validation set/testing set: 68%/17%/15%.
- Optimiser: AdamW.
- Weight decay = 0.00005.
- Criterion of loss: SmoothL1Loss (with value of β = 0.7).
- Dropout: Not used.

K-Fold cross-validation was applied during the training of the DNN [33]. For each DNN, 5 folds were assumed.

The Mean Absolute Error (MAE) was assumed to be minimised during the validation. After the training, the DNN from the fold with the lowest testing MAE was saved and used in the subsequent stages of the analysis.

MAE, Root Mean Square Error (RMSE), and coefficients of determination $R^{2}$ were computed during the creation of DNNs, according to the following equations:

(14) $$\mathrm{MAE}=\frac{1}{n}\sum_{i=1}^{n}\left|\Omega_{FEM,i}-\Omega_{DNN,i}\right|,$$

(15) $$\mathrm{RMSE}=\sqrt{\frac{1}{n}\sum_{i=1}^{n}{\left(\Omega_{FEM,i}-\Omega_{DNN,i}\right)}^{2}},$$

and

(16) $$R^{2}=1-\frac{\sum_{i=1}^{n}{\left(\Omega_{FEM,i}-\Omega_{DNN,i}\right)}^{2}}{\sum_{i=1}^{n}{\left(\Omega_{FEM,i}-\bar{\Omega_{FEM}}\right)}^{2}},$$

where *n* is the strength of the set (testing, validation, or training).

The architecture of the DNN (4 hidden layers, 60 neurons each) was selected empirically based on a series of preliminary tests. Table 3 presents these preliminary trials. Various configurations were evaluated, especially deep architectures (e.g., 3, 4, 5, and 10 hidden layers) with different neuron counts (e.g., 40, 50, 60, 70, 100).

#### 2.5.4. Genetic Algorithm Optimisation

The prepared DNN for each variant of boundary conditions was utilised as the objective function in single-criterion optimisation using the genetic algorithm (GA).

For all 11 boundary condition variants, separate GA scripts were prepared.

The following GA parameters were assumed:

- Library: geneticalgorithm;
- Decision variables: 4 lamination angles;
- Variable type: Integer, based on the discrete set of angles $\{-90^{\circ }$, $-85^{\circ }$, …, $90^{\circ }$};
- Maximum number of iterations: 140;
- Population size: 80;
- Mutation probability: 0.1;
- Elitism ratio: 0.08;
- Crossover probability: 0.85;
- Parents portion: 0.4;
- The maximum number of iterations without improvement: 25;
- Crossover type: Uniform;
- Number of GA trials: 50;
- Random seed: Unused.

For all boundary condition variants, GA was invoked 50 times. From the saved results, duplicates were removed, and the distinct solutions were verified using the same FEM (PyMAPDL) script used to prepare patterns for the DNN. After verification, the optimal set of angles corresponding to the highest value of Ω was selected and saved.

#### 2.5.5. Sensitivity Analysis

The next part of the analysis was to conduct the sensitivity analysis, which means to establish which variable (or variables), $\lambda_{1}$, $\lambda_{2}$, $\lambda_{3}$, or $\lambda_{4}$, affects the Ω value the most.

For the purpose of this stage of research, the elementary effects (EEs) [34] method was applied.

The elementary effects method provides an efficient approach for identifying the most influential input factors among the many present in a model. Morris [34] introduced the concept of elementary effects in 1991, proposing two sensitivity measures to determine whether input factors exhibit negligible, linear and additive, or nonlinear and interactive effects [35].

The EEs are calculated according to the following equation:

(17) $$EE_{i}=\frac{\left[Y\left(X_{1},X_{2},\ldots ,X_{i-1},X_{i}+\Delta ,\ldots X_{k}\right)-Y\left(X_{1},X_{2},\ldots ,X_{k}\right)\right]}{\Delta },$$

where

(18) $$\Delta =\frac{p}{2(p-1)},$$

where *p* is the number of levels of grid [35] and

(19) $$X=(X_{1},X_{2},X_{3},X_{4}).$$

Morris proposed the sensitivity measures μ and σ, where μ indicates the mean, and σ is the standard deviation of the set of elementary effects [35].

The mean μ indicates the overall factor’s influence on the output value (in this research, the output value is Ω). The standard deviation σ quantifies the aggregate effects of the factor, encompassing nonlinearities and interactions with other factors [35].

It means that the higher the value of μ, the stronger the influence of the factor. In addition, the low value of σ indicates that the values among the elementary effects are similar [35].

However, in 2007, Campolongo et al. [36] proposed the other approach. In this modified approach, the μ value was replaced by $\mu^{*}$ value, which is defined as the absolute values of the elementary effects’ mean.

The μ, μ^*^, and σ are calculated according to the following equations:

(20) $$\mu_{i}=\frac{1}{r}\sum_{j=1}^{r}EE_{i}^{j},$$

(21) $$\mu_{i}^{*}=\frac{1}{r}\sum_{j=1}^{r}\left|EE_{i}^{j}\right|,$$

and

(22) $$\sigma_{i}^{2}=\frac{1}{r-1}\sum_{j=1}^{r}{\left(EE_{i}^{j}-\mu \right)}^{2},$$

where $EE_{i}^{j}$ indicates the elementary effects relative to factor *i* computed along trajectory *j* [9] and *r* is the number of computed trajectories.

The elementary effects method requires preparing the samples. Every sample in this method is a trajectory.

The construction of trajectories is described in [9].

**Figure 2:**
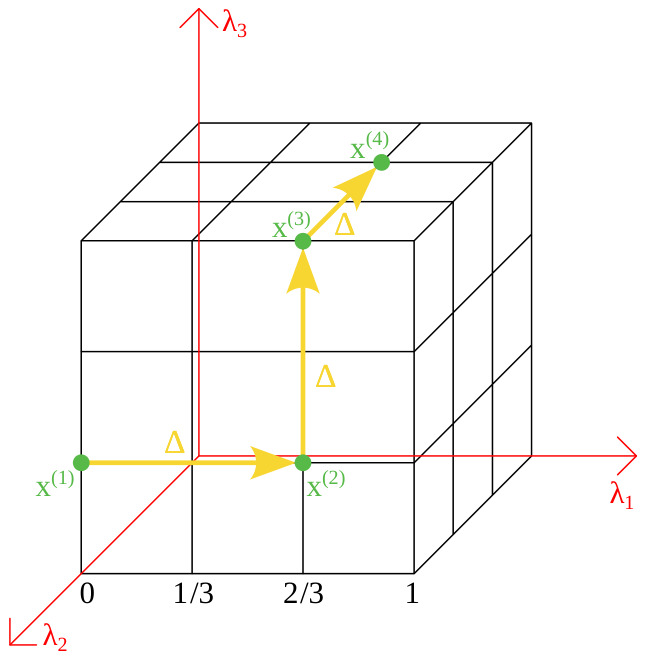
The way of creating the trajectory for k=3. The points of the trajectory are marked in green and the arrows indicating the proper steps of defining the trajectory are marked in yellow.

The example of creating the trajectory for k=3 is shown in Figure 2.

Trajectory construction begins with the random selection of a base vector $x^{*}$ from the *p*-level grid. The base vector $x^{*}$ functions solely as a seed for generation and is excluded from the final trajectory. The initial sampling point, $x^{\left(1\right)}$, is obtained by shifting one or more components of the base vector by a step size Δ. Subsequent points are generated sequentially using a one-at-a-time approach. The second point, $x^{\left(2\right)}$, is produced from $x^{\left(1\right)}$ by modifying exactly one randomly selected component *i*, where

(23) i∈{1,…,k}.

This modification consists of either adding or subtracting Δ

(24) $$x^{\left(2\right)}=x^{\left(1\right)}\pm e_{i}\Delta .$$

The subsequent point, $x^{\left(3\right)}$, is generated in a similar manner but must differ from $x^{\left(2\right)}$ in a unique component *j*, where j≠i. This process continues until the final point $x^{\left(k+1\right)}$ is obtained. The resulting design produces (k+1) points, with each consecutive sample differing by only one dimension, thereby ensuring that every component of the base vector $x^{*}$ is adjusted by Δ at least once [35].

In this study, a model with k=4 independent inputs was considered, varying within the four-dimensional unit cube across p=37 selected levels. Thus, the input space is discretised into a 37-level grid.

In addition, in this case, during the sensitivity analysis, the Sensitivity Analysis Library (SALib) [37] was applied and the values $\mu^{*}$ and σ were computed. Additionally, for each variant of boundary conditions, the number of trajectories r=500 was applied.

#### 2.5.6. Optimisation with Consideration of Uncertainties

The next stage of analysis was the optimisation of square composite slabs with consideration of uncertainties in the angle which affects the Ω value the most—$\lambda_{j,nom}$, where *j* indicates the index of the most influential angle, chosen after the SA (see Section 2.3). Although in real-world manufacturing environments uncertainties occur across multiple slab parameters, this approach considers them solely in the most influential angle, based on the SA results and the fact that assuming uncertainties in the less important angles does not significantly affect the optimisation results.

Initially, the new set of 2000 patterns for three selected boundary condition variants was prepared, where only the lamination angle $\lambda_{j,nom}$ was chosen randomly from the following continuous set:

(25) $$[-90^{\circ },90^{\circ }].$$

The new set of patterns for each selected boundary condition variant was necessary because, in the pattern sets generated according to Section 2.5.2, all four lamination angles were optimised. During optimisation with uncertainties, only one angle is optimised. Hence, new pattern sets are required.

For the purposes of optimisation with consideration of uncertainties, the following boundary condition variants were considered: SSSS, CCCC (symmetrical boundary condition configurations), and SCFF (unsymmetrical boundary condition configuration).

In the uncertainty-based optimisation, the remaining angles were held constant at the values obtained from the deterministic optimisation. This choice allows for isolating and studying the effect of perturbations in the angle $\lambda_{j,nom}$, while keeping the remaining parameters consistent with the high-performance lay-ups identified in the earlier part of the study.

The parameters of FEM simulations, the features of the applied computational server, and the stages of pattern generation are identical to the description presented previously.

The second stage of the optimisation with consideration of uncertainties, was preparing the surrogate models, the DNNs.

The DNNs were trained separately for each boundary condition variant. The parameters for each neural network are identical to those described in Section 2.5.3, apart from the architecture of the DNNs. For the purposes of preparing the surrogate models for this stage of analysis, the following architecture was assumed:

- Number of hidden layers: 5;
- Number of neurons in each hidden layer: 50.

The architecture of the DNN (5 hidden layers, 50 neurons each) was selected empirically based on a series of preliminary tests. Table 4 presents these preliminary trials.

Various configurations were evaluated, including the following architectures (3, 4, 5, and 6 hidden layers) with different neuron counts (40, 50, and 60).

K-Fold cross-validation was applied during the training of the DNN [33]. For each DNN, 5 folds were assumed.

Validation MAE was assumed to be minimised in each fold. The surrogate model with the lowest MAE on the test set was selected for subsequent analysis.

The prepared DNNs for the above-mentioned boundary condition variants were utilised as the objective function in single-criterion optimisation using the GA.

The GA parameters are identical to those described in Section 2.5.4, except for the number of decision variables and the variable type. For the purposes of the optimisation with consideration of uncertainties, the following types of parameters were assumed:

- Decision variable: Lamination angle, which affects the Ω value the most;
- Variable type: Real.

For all selected boundary condition variants, GA was invoked 50 times. During the first iteration of the GA script:

- The population of 80 $\lambda_{j,nom}$ angles is chosen randomly;
- Subsequently, 200 values of deviations are chosen randomly and added to the drawn by lot $\lambda_{j,nom}$ angles;
- For each set of uncertain angles (80 different sets, 200 uncertain angles each), 200 Ω values are computed and the 5% quantile of Ω values;
- Later, analysed $\lambda_{j,nom}$ angles undergo selection, crossover, and mutation.

The subsequent iterations are executed solely with the angle values, for which the highest 5% Ω quantiles were computed and their offspring were obtained.

After the GA optimisation, the $\lambda_{j,nom}$ angle corresponding to the highest value of the 5% quantile of Ω was chosen for the FEM verification with the use of the same FEM (PyMAPDL) script, which was used for preparing patterns for the DNNs. In this stage, 200 FEM simulations were executed, where at every execution, a different value of deviation was chosen randomly. After the verification, based on the obtained 200 Ω values, the value of the 5% quantile of Ω was computed as the solution of the optimisation with uncertainties problem.

## 3. Results and Discussion

In this section, the results of the following stages are presented: preparation of the DNNs, deterministic optimisation, sensitivity analysis, and optimisation with consideration of uncertainties.

### 3.1. Deep Neural Networks

For each variant of boundary conditions, a separate DNN was constructed. In addition, after every fold, the following diagrams were developed:

- Comparison of Ω values coming from FEM and DNN for the testing dataset;
- Value of training MAE depending on the number of epochs.

**Figure 3:**
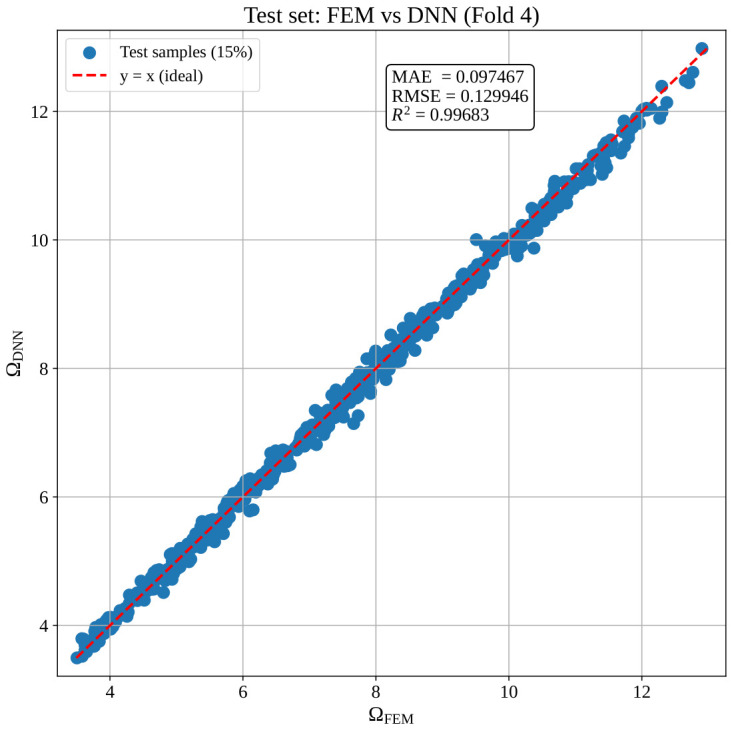
Comparison of the Ω values coming from neural network (vertical axis) and FEM (horizontal axis); the number of the fold with the lowest validation MAE error: 4, testing dataset.

**Figure 4:**
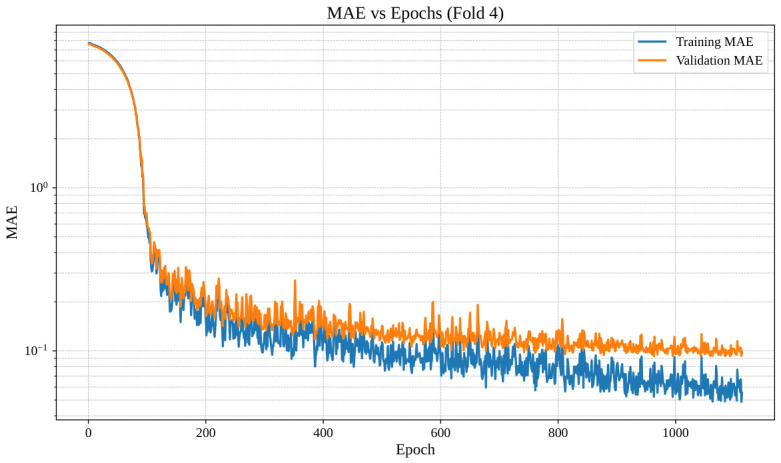
Graph of the MAE value depending on the number of epoch; the number of the fold with the lowest validation MAE error: 4.

The exemplary diagrams (for the variant CFFF) are shown in Figure 3 through Figure 4. For the other boundary condition variants, the surrogate model was trained analogously.

It is observed that the surrogate model is well prepared and a proper substitute for FEM computations, as the analysed blue points are located near and along the red straight line y = x.

The diagram presented in Figure 4 shows that there are very low fluctuations for the validation MAE from about epoch number 800 to the end of the learning DNN process. In addition, it could be noticed that early stopping was applied, which is seen on the right side of the diagram—the learning was ended near epoch number 1150 for this boundary condition variant.

### 3.2. Single-Criterion Deterministic Optimisation (GA)

A comparison of results coming from the DNN + GA approach and Yang [8] is presented in Table 5.

After scrutinizing Table 5, we can infer that there is no variant of the boundary conditions for which the sets of lamination angles obtained from Yang [8] and the DNN + GA approach are identical. The highest value of Ω was obtained for the variant CCCC, and the lowest value was observed for the variant CFFF. For the variant SCFF, the results of the Ω value coming from both methods (Yang [8] and DNN + GA) were the same.

Hence, for each boundary condition variant, there are different lamination schemes for which the Ω values are comparable.

It could also be noticed that values of the lamination angle λ_1_, obtained by Yang [8] and with the use of the DNN + GA approach, are identical (for eight boundary condition variants) or similar (for CCFF and SSCC cases). In the case of the lamination angle λ_2_, similar conclusions could be reached as in the case of the angle λ_1_. These observations are strongly related to the SA results, which are discussed in the subsequent section. The exception is the CCCC variant (λ_1_ = $90^{\circ }$ in Yang [8] vs. $0^{\circ }$ from the DNN + GA approach). This phenomenon could be due to the fact that, in this boundary condition case, the bisymmetry occurs in terms of the slab’s geometry and boundary conditions. In addition, values of $90^{\circ }$ and $-90^{\circ }$ for the lamination angle mean the same fibre orientation after rotation of the whole structure by $90^{\circ }$. Therefore, the difference between the lamination angles λ_1_, λ_2_, and λ_3_, in the solutions [90/0/0/90]_s_ (from Yang [8]) and [0/90/−90/−30]_s_ (from the DNN + GA approach) is 90 degrees, indicating a symmetric case and very similar Ω values. In the above-mentioned lamination schemes, the only significant difference occurred in the lamination angle value λ_4_ ($90^{\circ }$ vs. $-30^{\circ }$), which is the least important variable, according to the sensitivity analysis.

The percentage errors are collated in Table 6.

It is noticed that for some variants, optimal Ω values were slightly different than the Ω values from Yang [8], but the differences did not exceed 0.50%. The highest value of error was seen for the variant SCSF (0.50%), and the lowest value of non-zero error occurred for the variant SCSC (0.01%).

Table 7 shows the number of distinct solutions verified with the use of FEM for each variant of boundary conditions.

Table 7 shows that for the variant CCFF, the genetic algorithm pointed out 28 possible extremes, which were verified in the subsequent step of the analysis. For the variants SSCC, SSSC, and CCCC, the number of distinct results exceeded 20. For the variants SSSS and SCFF, the number of distinct results was 16 and 14, respectively. For the other variants, there were fewer than 10 trials, which were verified using FEM. The fact that multiple distinct FEM-verified solutions were obtained for each boundary condition configuration indicates that the adopted neural network exhibited adequate generalisation capability and did not impose significant exploratory limitations on the genetic algorithm.

A larger number of unique solutions obtained from independent runs of the genetic algorithm indicates the existence of a broad region of quasi-optimal solutions. This means that the objective function near the optimum may be relatively flat, and different configurations of the design variables may yield comparable responses. Conversely, a smaller number of recurring solutions suggests more clearly localised extrema, for which the algorithm more frequently converges to the same design configurations.

Statistical results: means, standard deviations, and 95% confidence intervals (CIs) of the FEM-verified Ω values are collated in Table 8.

The widest CI occurred for the variants SSSC, SSCC (the CI width is bigger than 0.1), and SSSS and CCCC (the CI width is about 0.1). For the other variants, the CIs are narrow and do not exceed 0.05.

It is observed that for the variant SSSC, the FEM-verified Ω values are sparser than for any other variant. The lower standard deviation values (between 0.146 and 0.207) occurred for four variants: CCCC, SCSF, SSCC, and SSSS. For the other variants, the Ω values are distributed close to the mean value.

Additionally, there is a relationship among the width of the CI, the standard deviation, and the number of distinct solutions in Table 7. For the boundary condition variants with the most distinct solutions, the standard deviation values and CI widths are the highest. The exception is the variant CCFF: even though there are 28 distinct solutions, the standard deviation is approximately 0.05, and the CI width is small.

### 3.3. Sensitivity Analysis

**Figure 5:**
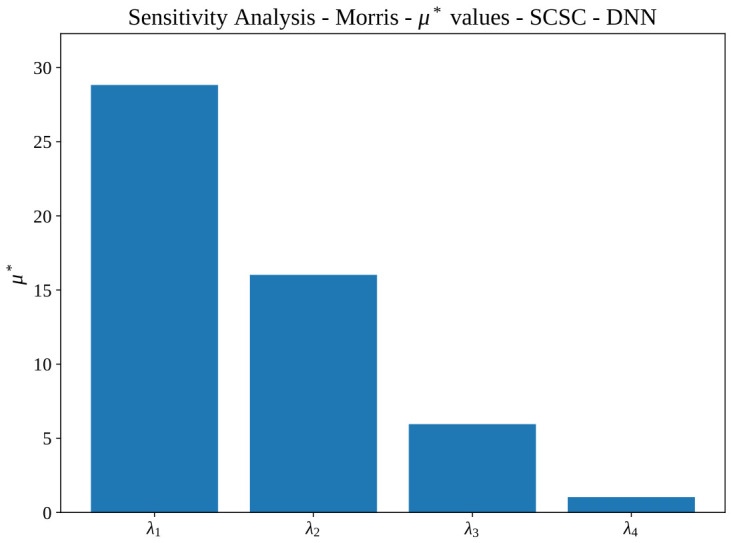
The μ^*^ results in the form of a bar chart. The higher the mean μ^*^, the higher the overall influence on the factor on the Ω value.

**Figure 6:**
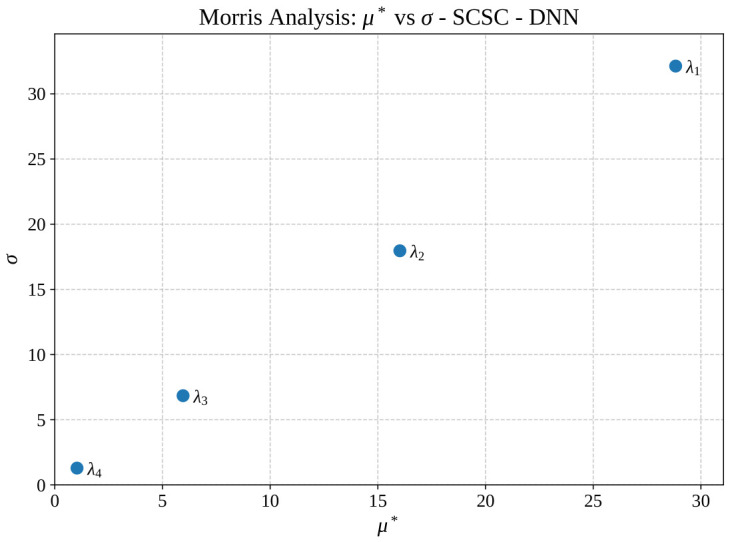
The μ^*^-σ diagram, presented as a scatter plot. The standard deviation σ measures the ensemble’s sensitivity to the effects of the factor, encompassing nonlinear influences and interactions with other factors [35].

All sensitivity analysis results in this subsection are presented for the variant SCSC in Figure 5 through Figure 6. The μ^*^ results bar chart and the μ^*^-σ plot are shown, respectively.

For the other boundary condition variants, the analysis was conducted analogously, and the results are collated in Table 9.

From the results collated in Table 9, we can notice that for each variant of the boundary conditions, the value of μ^*^ for the angle λ_1_ is the highest (in the range of 4.879 to 44.648) and for the angle λ_4_, it is the lowest (values in the range between 0.385 to 2.177). It means that angle λ_1_ primarily affects the Ω value. The angle λ_2_ is also a significant variable during the design of this composite slab, because the values of the μ^*^ parameter were lower than in the case of the angle λ_1_, but higher than values for the angles λ_3_ and λ_4_. The variables λ_3_ and λ_4_ are the least relevant variables.

The above results correspond to the data collated in Table 5. For example, for the variants SCSF and SSSS, the significant change in the lamination angle λ_4_ causes slight changes in the Ω value: 0.50% and 0.17%, respectively.

In addition, after analysing the data collated in Table 9, the other relationship between μ^*^ and σ is visible. A positive correlation is observed between the distance of the layer from the mid-plane and the values of parameters μ^*^ and σ determined for the lamination angle of this layer.

Hence, if the variable is more relevant, the effect of this variable is more nonconstant, which means, in some instances, a change in this parameter significantly impacts the output, whereas in others, the impact is minimal (depending on the point on the curve or the values of other factors).

### 3.4. Optimisation with Consideration of Uncertainties

After the sensitivity analysis, it was noticed that in all boundary condition variants, the angle λ_1_ affects the Ω value the most due to the highest μ^*^ value coming from the SA, as presented above. Hence, in this stage, the $\lambda_{1,nom}$ was optimised.

Table 10 shows the results of the optimisation with consideration of uncertainties for the $\lambda_{1,nom}$ lamination angle for three cases of boundary conditions, mentioned in Section 2.5.6.

Table 10 points out that the optimal lamination angle $\lambda_{1,nom}$ has a negative value solely in the case of the SSSS variant. In addition, the differences between the obtained 5% quantiles coming from DNN and FEM are slight (between 0.04% for the variant CCCC to 0.32% for the variant SCFF).

The comparison of λ_1_ values coming from optimisation without and with the inclusion of uncertainties is presented in the Table 11. In addition, values of Ω and 5% quantiles of Ω values are collated in this table.

From the Table 11, it could be noticed that the values of angles λ_1_ obtained from deterministic optimisation and rounded to the nearest integer coming from optimisation with uncertainties are the same. It means that values of the lamination angles λ_1_, computed from the deterministic optimisation, will also be optimal values in case of some deviations occurring in the lamination angle in this layer.

In addition, according to Table 10 through Table 11, the values of differences for the slab with unsymmetric configuration of boundary conditions (SCFF) are the highest (0.32% and 0.71%, respectively). These differences are lower for the slab with symmetric boundary conditions (CCCC and SSSS).

### 3.5. Duration of the Particular Analysis Stages per Boundary Condition Variant

Table 12 presents the duration of the particular analysis stages per boundary condition variant.

Table 12 indicates that the analysis times for each variant of the boundary conditions differ. The shortest time was observed for the CCCC variant (less than 100 h). For the other variants, this analysis time exceeded 4 days. The reader can see that the time spent generating the patterns (the second column of Table 12) accounts for more than 99% of the overall analysis time for each boundary condition case. The remaining stages account for less than 1% of the overall analysis time.

The DNN training time depends on the boundary condition variant and ranges from about 170 s (about 3 min) to about 310 s (more than 5 min). The time required for optimisation using GA, combined with FEM verification, is strongly correlated with the number of unique results that require FEM verification. The greater the number of unique trials, the longer this stage lasts.

The application of the surrogate model in the GA optimisation is significantly time-saving, because, according to the last two columns of Table 12, if the GA optimisation with FEM simulations was applied, the duration of the whole optimisation process would be approximately 1.6 years (80 chromosomes in population · 140 iterations per trial · 50 GA trials · 90 s per single FEM simulation).

Table 13 presents the overall times of the particular stages of the optimisation with the inclusion of uncertainty for the selected cases of boundary conditions.

The sensitivity analysis was the fastest stage because of the application of DNNs. The duration of this stage never exceeded 1 s. The SA time was computed for all 11 boundary condition variants; however, only the values for the SCFF, SSSS, and CCCC variants are shown in Table 13. If we used the FEM computations instead of the DNNs in the SA, the duration of the SA stage would be equal to approximately 62.5 h (5 Ω values per trajectory · 500 trajectories · 90 s per single FEM simulation). Therefore, the SA approach is significantly time-saving.

In the case of optimisation with the inclusion of uncertainties, according to Table 13, the longest stage is related to generating patterns for the purposes of preparing the surrogate models (more than 50 h for each case of boundary conditions), which represents between 91.23% and 92.12% of the overall analysis time. The DNNs’ training times are equal between about 1.5 and 3 min, which constitutes less than 0.10% of the total analysis time for each variant of boundary conditions. In addition, the time for GA optimisation and FEM verification ranges between about 4.24 and 5.24 h, accounting for between 7.80% and 8.72% of the total analysis time.

In addition, the application of the surrogate model also in the GA quantile-based optimisation is notably time-saving, because, according to the last two columns of Table 13, if the GA optimisation with FEM simulations was applied, the duration of the whole quantile-based optimisation process would significantly exceed 100 years (80 sets of chromosomes · 200 chromosomes each set · 140 iterations per trial · 50 GA trials · 90 s per single FEM simulation).

The proposed optimisation approach, which accounts for uncertainties, was also applied to an unsymmetrical slab.

### 3.6. Results for the Trapezium Composite Slabs

**Figure 7:**
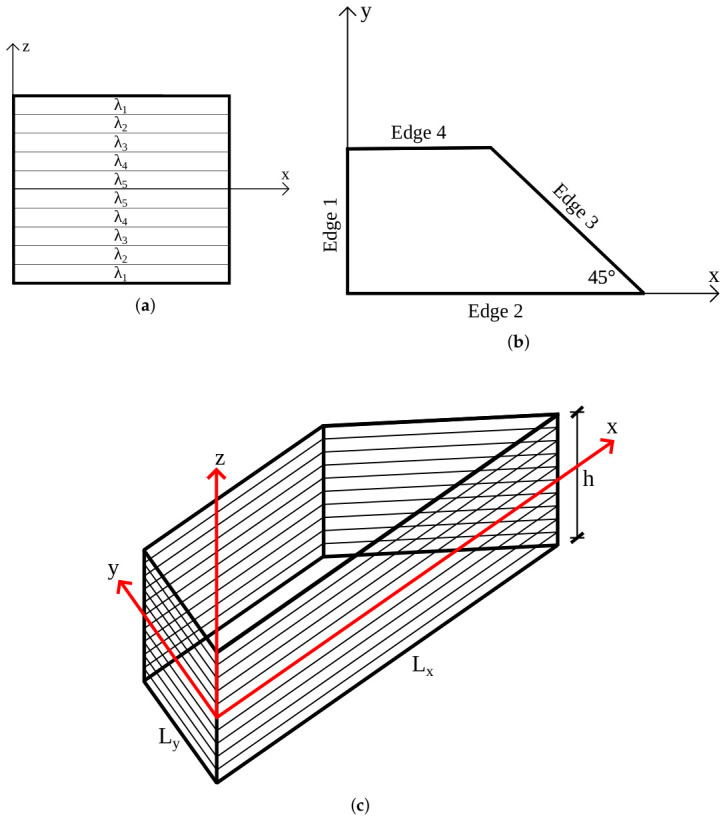
The scheme of the analysed composite trapezium slab. (**a**) Sequence of layers with indications of angles. (**b**) Numeration of edges. (**c**) 3D view of the analysed trapezium slab and the assumed coordinate system marked in red.

The DNN + GA approach and the optimisation of the 5% quantile of the Ω value were also applied to optimise the trapezium slabs with the inclusion of uncertainties for the angle $\lambda_{1,nom}$. The analysed trapezium slab comes from Yang [8] and is shown in Figure 7.

The dimensions, thickness, and material parameters of the trapezium slabs were assumed according to the descriptions in Yang [8] and are collated in Table 14.

As before, for the purposes of optimisation with consideration of uncertainties, the following boundary condition variants were considered: SSSS, CCCC (symmetrical configurations), and SCFF (unsymmetrical configuration).

Two variants of the lamination schemes were considered:

- $\left[\lambda_{1,nom}/0/0/0/0\right]$,
- $\left[\lambda_{1,nom}/90/90/90/90\right]$.

The DNNs were trained separately for each boundary condition variant. The parameters for each neural network are identical to those described in Section 2.5.3, apart from the architecture of the DNNs. For the purposes of preparing the surrogate models for this stage of analysis, the following architecture was assumed:

- Number of hidden layers: 6;
- Number of neurons in each hidden layer: 50.

The architecture of the DNN (six hidden layers, 50 neurons each) was selected empirically based on a series of preliminary tests.

The results for the trapezium slabs are presented in Table 15, Table 16, Table 17 and Table 18. Table 15 through Table 16 show the results of the optimisation with consideration of uncertainties for three cases of the above-mentioned boundary conditions for the trapezium slabs for the variant with constant angles equal to 0° and 90°, respectively.

Table 15 points out that the optimal lamination angles $\lambda_{1,nom}$ for all analysed boundary condition cases are close to 90°. In addition, the differences between the 5% quantiles obtained from DNN and FEM range from 0.16% to 0.56%.

Table 16 indicates that for the variant with constant angles equal to 90°, it could be concluded that the optimal lamination angles $\lambda_{1,nom}$ for all boundary condition variants are positive. For the SSSS and SCFF variants, the orientation of the fibres in the outermost layer related to the mid-surface of the slab is the same. In addition, the differences between the obtained 5% quantiles coming from DNN and FEM range between 0.05% and 0.10%. Table 17 through Table 18 present the overall times of optimisation with the inclusion of uncertainty stages for the trapezium slabs for the variant with constant angles equal to 0° and 90°, respectively.

In the case of optimisation with the inclusion of uncertainties, according to Table 17, the longest stage is related to the generating patterns for the purposes of preparing the surrogate models (between 10 and 11 h) for each case of boundary conditions, which represents between 90.27% and 91.27% of the general analysis time. The times of DNNs’ training are equal between about 1.5 and 3 min, which constitutes less than 0.54% of the total time of the analysis for each variant of boundary conditions. In addition, the time of GA optimisation and FEM verification ranges between about 1.02 and 1.04 h, which makes up between 8.51% and 9.20% of the total analysis time.

As above, according to Table 18, in the case of optimisation with the inclusion of uncertainties for the variant of the lamination scheme with constant angles equal to 90°, the longest stage for each case of boundary conditions is related to the generating patterns for the purposes of preparing the surrogate models (between 10.5 and 11.25 h), which represents between 90.44% and 90.64% of the general optimisation time. The times for the shortest stage, DNN training, are approximately 2–4 min, which constitute less than 0.57% of the total analysis time for each variant of boundary conditions. In addition, the GA optimisation and FEM verification times range between about 1.02 and 1.04 h, which makes up between 8.92% and 9.10% of the total analysis time.

In addition, the application of the surrogate model also in the GA quantile-based optimisation in the case of the trapezium slabs is notably time-saving, because, according to the last two columns of Table 17 through Table 18, if the GA optimisation with FEM simulations was applied, the duration of the whole quantile-based optimisation process would be about 106 years (80 sets of chromosomes · 200 chromosomes each set · 140 iterations per trial · 50 GA trials · 30 s per single FEM simulation).

The single FEM simulation time for the trapezium slabs is approximately 3 times shorter than in the case of the square slabs. The reasons for these differences are diverse areas of the analysed slabs (100 cm^2^ for the square slab vs. 37.5 cm^2^ for the trapezium slab; see Yang [8]) and, what is involved, the different number of finite elements in the analysed models.

## 4. Conclusions

This study presented a comprehensive framework for optimising the fundamental frequency of multi-layered composite slabs using a combination of FEM-generated datasets, DNN surrogate models, genetic algorithms, and Morris Sensitivity Analysis. The results obtained with the proposed DNN–GA approach are confirmed by those of Yang [8]. The sensitivity analysis revealed that λ_1_ is the dominant design variable for all analysed boundary conditions. It stems from the fact that the properties of layers located furthest from the mid-plane are most relevant in the design of composite structures. Additionally, the outer layers contribute most to the stiffness of the composite components.

The other key advantage of the proposed DNN–GA framework is computational efficiency: once the surrogate model is trained, sensitivity analysis, deterministic optimisation, and quantile-based optimisation require orders of magnitude less time than traditional FEM-based workflows, which could have lasted for even years.

Extending the optimisation to account for uncertainties demonstrated that robust solutions can be efficiently obtained by maximising the 5% quantile of Ω for both square and trapezium slabs. The results indicate that the proposed framework not only accelerates the optimisation process but also improves the reliability of laminate designs under realistic perturbations.

The FEM verification performed for both deterministic and uncertainty-aware optima confirms the accuracy and generalisation capability of the surrogate models. The proposed approach may be extended to more complex geometries and additional uncertain parameters, such as all lamination angles, thicknesses, material parameters, etc., in future research.

## Figures and Tables

**Table 1:** Parameters applied in Equation (5) through Equation (7), based on Yang [8].

Parameter	Meaning of the Parameter	Value of Parameter	Unit
Ω	non-dimensional frequency parameter	depending on the values of lamination angles	[-]
ω	the first natural frequency	as above	[rad/s]
*f*	frequency	as above	[Hz]
*a*	length of the slab edge	0.1	[m]
*h*	thickness of the slab	1	[mm]

**Table 2:** Parameters of the composite material, applied in the analysis, based on [8].

Parameter	Meaning of the Parameter	Value	Unit
ρ	density	1450	[kg/m^3^]
$E_{11}$	Young’s modulus in the 1-direction	138	[GPa]
$E_{22}$	Young’s modulus in the 2-direction	8.96	[GPa]
$G_{12}=G_{13}$	shear moduli (in-plane and transverse, respectively)	7.1	[GPa]
$G_{23}$	transverse shear modulus	6.21	[GPa]
$\nu_{12}$ = $\nu_{21}$	Poisson’s ratios	0.3	[-]

**Table 3:** The results of the preliminary tests for selecting the DNN architecture.

Number of Variant	Number of Hidden Layers	Number of Neurons (in Each Hidden Layer)	Testing MAE/Training MAE	R^2^-Testing Set	R^2^-Training Set
1	3	50	0.3782/0.2783	0.96934	0.98325
2	4	50	0.2761/0.2127	0.98543	0.99071
3	4	60	0.2223/0.1352	0.98982	0.99616
4	4	70	0.3565/0.1554	0.97254	0.99488
5	5	60	0.3386/0.1735	0.97442	0.99329
6	5	70	0.3014/0.1513	0.98044	0.99511
7	10	60	0.4656/0.4396	0.95925	0.96122
8	10	100	0.2880/0.2600	0.98436	0.98662
9	5	50	0.3293/0.1724	0.97363	0.99316
10	5	40	0.3172/0.1951	0.97949	0.99181

**Table 4:** The results of the preliminary tests for selecting the DNN architecture—optimisation with consideration of uncertainties.

Number of Variant	Number of Hidden Layers	Number of Neurons (in Each Hidden Layer)	Testing MAE/Training MAE	R^2^-Testing Set	R^2^-Training Set
1	3	50	0.0697/0.0691	0.99712	0.99720
2	4	50	0.0634/0.0674	0.99801	0.99779
3	4	60	0.0589/0.0622	0.99830	0.99811
4	5	50	0.0418/0.0437	0.99907	0.99899
5	5	60	0.0550/0.0558	0.99857	0.99853
6	6	40	0.0615/0.0638	0.99790	0.99774
7	6	50	0.0467/0.0467	0.99905	0.99903

**Table 5:** Comparison of Ω values coming from Yang [8] and the DNN + GA approach.

Boundary Conditions	Lamination Scheme from the Article [8]	Ω Value from the Article [8]	Lamination Scheme from DNN + GA Approach	Ω Value from DNN + GA Approach
CFFF	${\left[0/0/0/0\right]}_{s}$	13.19	${\left[0/0/5/5\right]}_{s}$	13.17
SCFF	${\left[70/-45/70/75\right]}_{s}$	15.61	${\left[70/-45/65/-50\right]}_{s}$	15.61
CCFF	${\left[50/-40/30/-85\right]}_{s}$	18.09	${\left[45/-40/45/40\right]}_{s}$	18.08
CFCF	${\left[0/0/0/0\right]}_{s}$	83.57	${\left[0/-5/0/10\right]}_{s}$	83.34
SSSF	${\left[0/0/0/0\right]}_{s}$	38.05	${\left[0/0/-10/35\right]}_{s}$	37.92
SCSF	${\left[0/0/0/0\right]}_{s}$	38.48	${\left[0/0/0/75\right]}_{s}$	38.29
SSSS	${\left[-45/45/45/45\right]}_{s}$	53.06	${\left[-45/45/45/-45\right]}_{s}$	52.97
SSSC	${\left[65/-60/-60/-70\right]}_{s}$	63.31	${\left[65/-55/-65/-45\right]}_{s}$	63.20
SCSC	${\left[90/90/90/90\right]}_{s}$	86.52	${\left[90/-90/90/90\right]}_{s}$	86.51
SSCC	${\left[45/-45/-50/-35\right]}_{s}$	68.38	${\left[50/-5/-45/-65\right]}_{s}$	68.29
CCCC	${\left[90/0/0/90\right]}_{s}$	89.32	${\left[0/90/-90/-30\right]}_{s}$	89.29

**Table 6:** The percentage errors between the Ω values coming from Yang [8] and the DNN + GA approach.

Boundary Conditions	Ω Value from the Article [8]	Ω Value from DNN + GA	Difference [%]
CFFF	13.19	13.17	0.15
SCFF	15.61	15.61	0.00
CCFF	18.09	18.08	0.06
CFCF	83.57	83.34	0.28
SSSF	38.05	37.92	0.34
SCSF	38.48	38.29	0.50
SSSS	53.06	52.97	0.17
SSSC	63.31	63.20	0.17
SCSC	86.52	86.51	0.01
SSCC	68.38	68.29	0.13
CCCC	89.32	89.29	0.03

**Table 7:** The number of distinct solutions verified with the use of FEM after the GA optimisation.

Boundary Conditions	Distinct Solutions Verified with the Use of FEM After the GA
CFFF	4
SCFF	14
CCFF	28
CFCF	4
SSSF	7
SCSF	7
SSSS	16
SSSC	21
SCSC	8
SSCC	23
CCCC	26

**Table 8:** Statistical analysis of Ω—FEM verification.

Boundary Conditions	Number of Samples	Mean Value	Standard Deviation	95% CI
CCCC	50	89.022	0.175	(88.972, 89.072)
CCFF	50	18.004	0.051	(17.990, 18.019)
CFCF	50	83.031	0.045	(83.018, 83.043)
CFFF	50	13.138	0.043	(13.126, 13.151)
SCFF	50	15.535	0.087	(15.510, 15.560)
SCSC	50	86.483	0.030	(86.474, 86.491)
SCSF	50	38.115	0.146	(38.073, 38.156)
SSCC	50	68.085	0.207	(68.026, 68.144)
SSSC	50	62.769	0.481	(62.632, 62.906)
SSSF	50	37.812	0.047	(37.799, 37.826)
SSSS	50	52.648	0.170	(52.600, 52.696)

**Table 9:** The overall results of the sensitivity analysis.

Boundary Conditions	$\mu^{*}$ of $\lambda_{1}$	$\mu^{*}$ of $\lambda_{2}$	$\mu^{*}$ of $\lambda_{3}$	$\mu^{*}$ of $\lambda_{4}$	σ of $\lambda_{1}$	σ of $\lambda_{2}$	σ of $\lambda_{3}$	σ of $\lambda_{4}$
CFFF	6.476	3.882	1.585	0.367	7.326	4.460	1.840	0.460
SCFF	5.784	3.616	1.468	0.403	6.504	4.152	1.768	0.517
CCFF	4.879	3.818	1.681	0.313	4.168	3.850	1.970	0.385
CFCF	44.648	24.034	9.562	1.742	49.755	27.508	11.384	2.177
SSSF	16.649	9.530	3.846	0.816	18.855	10.758	4.508	1.031
SCSF	12.692	8.067	3.204	0.727	14.387	9.148	3.798	0.935
SSSS	6.394	6.143	2.904	0.812	8.121	7.653	3.866	1.031
SSSC	13.701	9.052	3.776	0.836	15.279	10.425	4.484	1.080
SCSC	28.827	16.023	5.955	1.032	32.133	17.963	6.846	1.289
SSCC	6.213	6.041	2.585	0.646	7.982	7.687	3.506	0.841
CCCC	5.810	5.663	2.487	0.590	7.781	7.395	3.402	0.772

**Table 10:** The results of the quantile-based optimisation for selected boundary conditions.

Boundary Conditions	$\lambda_{1,nom}$ [°]	$Q_{05}(\Omega {)}_{\mathrm{DNN}}$	$Q_{05}(\Omega {)}_{\mathrm{FEM}}$	Difference Between Quantile Values [%]
CCCC	0.87	89.18	89.14	0.04
SSSS	−45.31	52.86	52.82	0.08
SCFF	70.59	15.55	15.50	0.32

**Table 11:** The comparison of chosen results coming from optimisation without and with the inclusion of uncertainties for selected boundary conditions.

Boundary Conditions	$\lambda_{1}$ from Table 5 [°]	$\lambda_{1,nom}$ Rounded to the Nearest Integer [°]	Ω from Table 5	$Q_{05}(\Omega {)}_{\mathrm{FEM}}$	Difference Between Ω Value and Quantile Value [%]
CCCC	0	0	89.29	89.14	0.17
SSSS	−45	−45	52.97	52.82	0.28
SCFF	70	70	15.61	15.50	0.71

**Table 12:** The duration of individual stages of the analysis, described in the syntax [hh:mm:ss].

Boundary Conditions	Time of Generating 4000 Patterns with the Use of PyMAPDL [hh:mm:ss]	Time of DNN Training [s]	Time of Running GA (50 Trials) with DNN and FEM Verification [s]	Total Time [hh:mm:ss]	Estimated Time of Running GA (50 Trials) with FEM [years]
CFFF	107:57:29	170.41	574.68	108:09:46	≈1.6
SCFF	106:09:14	253.60	1520.63	106:38:49	≈1.6
CCFF	104:20:37	270.51	2765.42	105:11:14	≈1.6
CFCF	104:27:04	299.52	560.20	104:41:24	≈1.6
SSSF	104:06:00	235.46	847.71	104:24:04	≈1.6
SCSF	103:49:09	241.89	842.86	104:07:14	≈1.6
SSSS	101:55:42	281.17	1642.39	102:27:46	≈1.6
SSSC	≈100 h ^*^	260.16	2103.72	≈100.5 h ^*^	≈1.6
SCSC	100:30:51	266.73	933.19	100:50:51	≈1.6
SSCC	100:41:35	307.19	1985.01	101:19:48	≈1.6
CCCC	94:11:38	304.03	2370.14	94:56:13	≈1.6

* Estimated values. Execution time for the generating patterns for the SSSC variant was not saved due to a technical error. This error does not affect the computational results.

**Table 13:** The duration of individual stages of the optimisation with inclusion of uncertainties, described in the syntax [hh:mm:ss].

Boundary Conditions	Time of the Sensitivity Analysis with the DNN [s]	Time of Generating 2000 Patterns with the Use of PyMAPDL [hh:mm:ss]	Time of DNN Training [s]	Time of Running GA (50 Trials) with DNN and FEM Verification [s]	Total Time [hh:mm:ss]	Estimated Time of GA Optimisation with FEM [Years]
SCFF	0.48	54:45:46	97.88	18,850.79	60:01:35	more than 100
SSSS	0.52	53:26:35	186.38	17,462.52	58:20:44	more than 100
CCCC	0.55	50:01:28	159.32	15,254.49	54:18:22	more than 100

**Table 14:** Parameters of the trapezium slabs, based on Yang [8].

Parameter	Meaning of the Parameter	Value of Parameter	Unit
$L_{x}$	length of the longer trapezium base	100	[mm]
$L_{y}$	height of the trapezium	50	[mm]
*h*	thickness of the slab	5	[mm]
ρ	density	2500	[kg/m^3^]
$\nu_{12}$ = $\nu_{21}$	Poisson’s ratios	0.25	[-]
$E_{11}$	Young’s modulus in the 1-direction	400	[GPa]
$E_{22}$	Young’s modulus in the 2-direction	10	[GPa]
$G_{12}$ = $G_{13}$ = $G_{23}$	shear moduli (in-plane and transverse, respectively)	6	[GPa]

**Table 15:** The comparison of chosen results coming from optimisation without and with the inclusion of uncertainties for selected boundary conditions—trapezium slabs—with constant angles equal to 0°.

Boundary Conditions	$\lambda_{1,nom}$ [°]	$Q_{05}(\Omega {)}_{\mathrm{DNN}}$	$Q_{05}(\Omega {)}_{\mathrm{FEM}}$	Difference Between Quantile Values [%]
CCCC	90.00	21.269	21.314	0.21
SSSS	83.73	13.540	13.518	0.16
SCFF	89.61	5.690	5.722	0.56

**Table 16:** The comparison of chosen results coming from optimisation without and with the inclusion of uncertainties for selected boundary conditions—trapezium slabs—with constant angles equal to 90°.

Boundary Conditions	$\lambda_{1,nom}$ [°]	$Q_{05}(\Omega {)}_{\mathrm{DNN}}$	$Q_{05}(\Omega {)}_{\mathrm{FEM}}$	Difference Between Quantile Values [%]
CCCC	11.51	20.205	20.222	0.08
SSSS	89.97	14.428	14.414	0.10
SCFF	89.92	6.057	6.060	0.05

**Table 17:** The duration of individual stages of the optimisation with the inclusion of uncertainties for the trapezium slabs—constant angles equal to 0°, described in the syntax [hh:mm:ss].

Boundary Conditions	Time of Generating 2000 Patterns with the Use of PyMAPDL [hh:mm:ss]	Time of DNN Training [s]	Time of Running GA (50 Trials) and FEM Verification [s]	Total Time [hh:mm:ss]	Estimated Time of GA Optimisation with FEM [Years]
SCFF	10:59:20	97.93	3687.55	12:02:25	≈106
SSSS	10:42:58	227.56	3675.51	11:48:01	≈106
CCCC	10:09:53	216.12	3729.79	11:15:39	≈106

**Table 18:** The duration of individual stages of the optimisation with inclusion of uncertainties for the trapezium slabs—constant angles equal to 90°, described in the syntax [hh:mm:ss].

Boundary Conditions	Time of Generating 2000 Patterns with the Use of PyMAPDL [hh:mm:ss]	Time of DNN Training [s]	Time of Running GA (50 Trials) and FEM Verification [s]	Total Time [hh:mm:ss]	Estimated Time of GA Optimisation with FEM [years]
SCFF	11:13:09	111.96	4056.44	12:22:37	≈106
SSSS	10:58:48	248.00	3896.35	12:07:52	≈106
CCCC	10:28:06	235.84	3748.30	11:34:30	≈106

## Data Availability

The original contributions presented in the study are included in the article, further inquiries can be directed to the corresponding authors.

## Abbreviations

The following abbreviations are used in this manuscript:

ABC	Artificial Bee Colony
CHP	Co-evolutionary Host–Parasite
CI	Confidence Interval
DNN	Deep Neural Network
EEs	Elementary Effects
FEM	Finite Element Method
GA	Genetic Algorithm
HWOA	Hybrid Whale Optimisation Algorithm
LOA	Layerwise Optimisation Approach
MAE	Mean Absolute Error
MAPDL	Mechanical Ansys Parametric Design Language
ReLU	Rectified Linear Unit
RMSE	Root Mean Square Error
RPSOLC	Repulsive Particle Swarm Optimisation with Local search and Chaotic perturbation
SA	Sensitivity Analysis
SALib	Sensitivity Analysis Library
